# Implications of Big Data Analytics, AI, Machine Learning, and Deep Learning in the Health Care System of Bangladesh: Scoping Review

**DOI:** 10.2196/54710

**Published:** 2024-10-28

**Authors:** Md Ashraful Alam, Md Refat Uz Zaman Sajib, Fariya Rahman, Saraban Ether, Molly Hanson, Abu Sayeed, Ema Akter, Nowrin Nusrat, Tanjeena Tahrin Islam, Sahar Raza, K M Tanvir, Mohammod Jobayer Chisti, Qazi Sadeq-ur Rahman, Akm Hossain, MA Layek, Asaduz Zaman, Juwel Rana, Syed Moshfiqur Rahman, Shams El Arifeen, Ahmed Ehsanur Rahman, Anisuddin Ahmed

**Affiliations:** 1 Maternal and Child Health Division International Centre for Diarrheal Disease Research, Bangladesh Dhaka Bangladesh; 2 Department of Health and Kinesiology University of Illinois Champaign and Urbana, IL United States; 3 Department of Women's and Children's Health Uppsala University Uppsala Sweden; 4 Department of Computer Science and Engineering Jagannath University Dhaka Bangladesh; 5 Faculty of Information Technology Monash University Melbourne Australia; 6 Department of Epidemiology, Biostatistics and Occupational Health McGill University Montreal, QC Canada; 7 Research and Innovation Division South Asian Institute for Social Transformation Dhaka Bangladesh

**Keywords:** machine learning, deep learning, artificial intelligence, big data analytics, public health, health care, mobile phone, Bangladesh

## Abstract

**Background:**

The rapid advancement of digital technologies, particularly in big data analytics (BDA), artificial intelligence (AI), machine learning (ML), and deep learning (DL), is reshaping the global health care system, including in Bangladesh. The increased adoption of these technologies in health care delivery within Bangladesh has sparked their integration into health care and public health research, resulting in a noticeable surge in related studies. However, a critical gap exists, as there is a lack of comprehensive evidence regarding the research landscape; regulatory challenges; use cases; and the application and adoption of BDA, AI, ML, and DL in the health care system of Bangladesh. This gap impedes the attainment of optimal results. As Bangladesh is a leading implementer of digital technologies, bridging this gap is urgent for the effective use of these advancing technologies.

**Objective:**

This scoping review aims to collate (1) the existing research in Bangladesh’s health care system, using the aforementioned technologies and synthesizing their findings, and (2) the limitations faced by researchers in integrating the aforementioned technologies into health care research.

**Methods:**

MEDLINE (via PubMed), IEEE Xplore, Scopus, and Embase databases were searched to identify published research articles between January 1, 2000, and September 10, 2023, meeting the following inclusion criteria: (1) any study using any of the BDA, AI, ML, and DL technologies and health care and public health datasets for predicting health issues and forecasting any kind of outbreak; (2) studies primarily focusing on health care and public health issues in Bangladesh; and (3) original research articles published in peer-reviewed journals and conference proceedings written in English.

**Results:**

With the initial search, we identified 1653 studies. Following the inclusion and exclusion criteria and full-text review, 4.66% (77/1653) of the articles were finally included in this review. There was a substantial increase in studies over the last 5 years (2017-2023). Among the 77 studies, the majority (n=65, 84%) used ML models. A smaller proportion of studies incorporated AI (4/77, 5%), DL (7/77, 9%), and BDA (1/77, 1%) technologies. Among the reviewed articles, 52% (40/77) relied on primary data, while the remaining 48% (37/77) used secondary data. The primary research areas of focus were infectious diseases (15/77, 19%), noncommunicable diseases (23/77, 30%), child health (11/77, 14%), and mental health (9/77, 12%).

**Conclusions:**

This scoping review highlights remarkable progress in leveraging BDA, AI, ML, and DL within Bangladesh’s health care system. The observed surge in studies over the last 5 years underscores the increasing significance of AI and related technologies in health care research. Notably, most (65/77, 84%) studies focused on ML models, unveiling opportunities for advancements in predictive modeling. This review encapsulates the current state of technological integration and propels us into a promising era for the future of digital Bangladesh.

## Introduction

### Background

In recent years, the global surge in digital technology has ushered in a data-driven decision-making era across diverse sectors, including the health care and public health sectors [1]. The convergence of state-of-the-art technologies, such as big data analytics (BDA), artificial intelligence (AI), machine learning (ML), and deep learning (DL), is collectively reshaping the health care and epidemiological landscape [2]. This paradigm shift promises to revolutionize the comprehension and management of health challenges, offering the potential for more efficient and effective interventions [2].

The health care system fundamentally revolves around safeguarding and enhancing individual and community health by integrating public health and medical care services, using health promotion, disease and injury prevention, infectious disease surveillance, response, and research. Interdisciplinary research now explores various factors influencing individual and community well-being, ranging from microbial infections to social determinants, behavioral patterns, and various facets of lifestyle [3]. In addition, the widespread adoption of emerging technologies such as telemedicine, clinical decision support systems, electronic health records, personal health records, and mobile health care have generated a vast reservoir of health-related data [4].

Harnessing the latent potential of these extensive data, digital technologies, such as BDA, AI, ML, and DL, can surpass conventional data management systems [5]. By applying AI algorithms, ML techniques, and DL capabilities, BDA offers invaluable support to clinicians, health care providers, and policy makers in intervention designing, planning, and execution; expediting disease detection; predicting health outcomes; and advancing personalized medicine [6]. This ultimately results in both cost-effectiveness and quality outcomes [7].

On a global scale, extensive health data can be categorized into 5 primary domains: biological measurements of participants (eg, genomic or metabolomic datasets), participant context measurements (eg, environmental variables), administrative medical records, participant tracking data from devices equipped with GPS, and electronic data from various sources (eg, social media or search history records) [8]. Using BDA, AI, ML, and DL algorithms to extract valuable insights from these datasets can significantly enhance individual health management and enable swift disease trend detection and outbreak responses [9]. Beyond health care, BDA, AI, and ML can play crucial roles in enabling molecular data analysis, synthesis of genetic and clinical information to craft personalized treatments, and development of predictive models based on omics data to identify genetic disease markers and develop effective novel drugs [10].

In Bangladesh, which is a densely populated South Asian nation with >160 million inhabitants, integrating modern technologies with the health care system holds immense potential. Bangladesh grapples with various health-related challenges, spanning from infectious diseases, noncommunicable diseases (NCDs) and maternal and child health issues to environmental health concerns [11]. To tackle these multifaceted challenges comprehensively, the government of Bangladesh (GoB) has actively adopted advanced technologies to elevate the quality of health care services. Initiatives such as District Health Information System 2, the open medical record system, and the open-source smart register platform have revolutionized health data collection and service delivery [12]. These automated applications are instrumental in generating specific health-related evidence across different tiers of health care services and facilitating need-based resource allocations. Although there are potential challenges regarding proper data governance and legal framework, the immense potential to use these data has been capturing the attention of interdisciplinary researchers and practitioners eager to explore the potential of BDA, AI, ML, and DL technologies to use this wealth of datasets [12].

### Objectives

In Bangladesh, instances of using BDA, AI, ML, and DL technologies in health care and public health research are sporadic. However, the dearth of clear evidence has led to an inadequate understanding of the proper application of these technologies and their potential contributions to health care and public health research [12]. This scoping review is motivated by the need to address this gap, systematically examining existing research that explores the use of BDA, AI, ML, and DL technologies to analyze health care data in Bangladesh. The primary objective was to compile and synthesize available research findings on these domains in the country’s health care sector. This includes identifying trends, patterns, and methodologies for applying BDA, AI, ML, and DL technologies. The secondary objective was to assess the limitations faced by researchers in integrating these technologies into health care research, aiming to provide insights into the challenges that may hinder their widespread adoption. Overall, the insights derived from this review aimed to furnish evidence-based strategies for policy makers, health care professionals, and researchers, ultimately contributing to enhancing health outcomes and the overall well-being of the Bangladeshi population. Moreover, this exploratory journey is guided by the goal of uncovering valuable insights that can significantly shape the future of health care, not only within Bangladesh but also on a global scale.

## Methods

### Overview

We have followed the standard guidelines for scoping reviews. This scoping review used the PRISMA-ScR (Preferred Reporting Items for Systematic Reviews and Meta-Analyses Extension for Scoping Reviews; Multimedia Appendix 1) guidelines in line with best practices for research paper selection and reporting [13], and its protocol has been registered with the Open Science Framework [14]. We have provided the PRISMA-ScR checklist.

### Ethical Considerations

There were no human participants in this review, and ethics approval is not applicable.

### Inclusion and Exclusion Criteria

We included all studies that are primarily focused on health care and public health issues in Bangladesh and used any of the cross-cutting technologies, such as BDA, AI, ML, and DL, in analyzing datasets. The studies that used publicly available global or web-based health care and public health datasets in their research but validated their findings in the context of Bangladesh were also included in this review. All journal articles and conference articles published between January 1, 2000, and September 10, 2023, written in English and publicly accessible, were included regardless of research design and sample sizes.

Studies conducted by Bangladeshi researchers but not focusing on the context of Bangladesh, review articles, book chapters, commentaries, gray literature, and articles published in other languages (other than English) were excluded from the study. Manuscripts that were not accessible through institutional credentials or after contacting the authors were also excluded.

A comparative textbox (Textbox 1) presents the inclusion and exclusion criteria.

**Inclusion and exclusion criteria.**

**Inclusion criteria**

- Article type: journal articles, conference articles
- Focus: studies primarily focused on public health issues in Bangladesh using big data analytics, artificial intelligence, machine learning, or deep learning
- Data source: studies using publicly available global or web-based public health datasets validated in the context of Bangladesh
- Language: English
- Publication date: between January 1, 2000, and September 10, 2023
- Accessibility: full text accessible through institutional access or direct contact with authors

**Exclusion criteria**

- Article type: review articles, book chapters, commentaries, gray literature
- Focus: studies not focusing on the context of Bangladesh
- Data source: studies using publicly available global or online public health datasets validated in other countries
- Language: papers published in languages other than English
- Publication date: articles published before January 2000 and after the database search date
- Accessibility: manuscripts not accessible through institutional access or direct contact with authors

### Data Sources and Literature Search

We conducted a search across 4 electronic databases: MEDLINE (via PubMed), IEEE Xplore, Scopus, and Embase, on September 10, 2023. Our search was refined by applying a date filter, limiting results from January 1, 2000, to the search date. To identify relevant information, we used a set of key search terms related to the population, concept, and context framework (Textbox 2).

**Key terms of the population, concept, and context framework for developing a comprehensive search strategy.**

**Population**

- Public health
- Community health
- Preventive medicine
- Epidemiology
- Health care management
- Public health and safety
- Population health
- Public Health Surveillance
- Public health informatics

**Concept**

- Big data analytics
- Artificial intelligence
- Machine learning
- Deep learning

**Context**

- Bangladesh
- Bangladesh health system

For each database, we meticulously crafted a comprehensive search strategy, tailoring it to the specific databases by incorporating both key terms and database-specific index terms (Multimedia Appendix 2).

The main search keywords included terms such as “Artificial Intelligence,” “AI,” “Machine Intelligence,” and “Cognitive Computing” for AI; “Machine Learning,” “Transfer Learning,” “Predictive Modeling,” and “Pattern Recognition” for ML; “Deep Learning,” “Neural Network,” “Artificial Neural Network,” and “Deep Neural Network” for DL; and “Big Data,” “Data Mining,” “Big Data Analytics,” and “Data Science” for BDA. In addition, we included keywords relevant to the health care context, such as “Healthcare System,” “Health Services,” “Clinical Settings,” and “Public Health,” and geographic context-specific terms, such as “Bangladesh,” “Bangladeshi,” and “Bangladesh Health System.”

Using these targeted keywords, we aimed to capture a comprehensive set of studies relevant to the use of BDA, AI, ML, and DL technologies within the health care system of Bangladesh.

### Screening Process

Following the retrieval of articles from the databases, they were imported into EndNote (version 9; Clarivate), and duplicates were removed. Subsequently, the review team was divided into 2 groups: group 1 and group 2. Each group was assigned a subset of articles from the total pool of retrieved articles (group 1: 826/1653, 49.97%; group 2: 827/1653, 50.03%) to independently screen the titles and abstracts in accordance with the established inclusion and exclusion criteria.

Each reviewer independently screened the titles and abstracts of their assigned articles and cross-checked their decisions within groups. After this initial screening, full-text screening was performed by 2 individual reviewers. Any disagreements or uncertainties that arose during any of the screening stages were promptly resolved through discussion and consensus among the reviewers. In cases of disagreements, a third reviewer stepped in to refute the disagreements, considering inclusion or exclusion criteria. This rigorous screening process ensured that the selected studies aligned with the defined objectives and inclusion or exclusion criteria, enhancing the robustness and reliability of this scoping review.

### Data Extraction, Analysis, and Quality Assessment

We developed a data extraction sheet to capture relevant data in line with the review objectives. The extraction variables comprised descriptive information of the included studies, such as title, author, published year, sample size, dataset information, study design, the objective of the study, the technology used, the domain of public health, results, limitations, and practical implementation of the study result. Following data extraction, we analyzed and categorized the data according to the dataset information, the similarity of the disease domains, methods used for analysis, and the performance of algorithms.

The quality of all the included studies was assessed following the Joanna Briggs Institute Critical Appraisal tool [15]. In total, 2 individual reviewers assessed the quality of the studies, and disagreement was solved upon discussion. For overall grading using the Critical Appraisal tool, we followed a published systematic review that used a rule of thumb for overall grading [16]. For case-control studies, if 0 to 3 “yes” answers were obtained out of 10 screening questions, the study was marked as poor quality. Consecutively, if 4 to 6 “yes” answers were obtained, the study was marked as moderate quality, and if >6 “yes” answers were obtained, the study was ranked as good quality. For cohort studies, among 11 screening questions, 0 to 3 “yes” answers were marked as poor quality, 4 to 7 “yes” answers as moderate quality, and >6 “yes” answers as good quality. For cross-sectional studies, among 8 screening questions, 0 to 3 “yes” answers were marked as poor quality, 4 to 5 “yes” answers as moderate quality, and >6 “yes” answers as good quality study (Multimedia Appendix 3).

## Results

### Selection of Sources of Evidence

**Figure 1:**
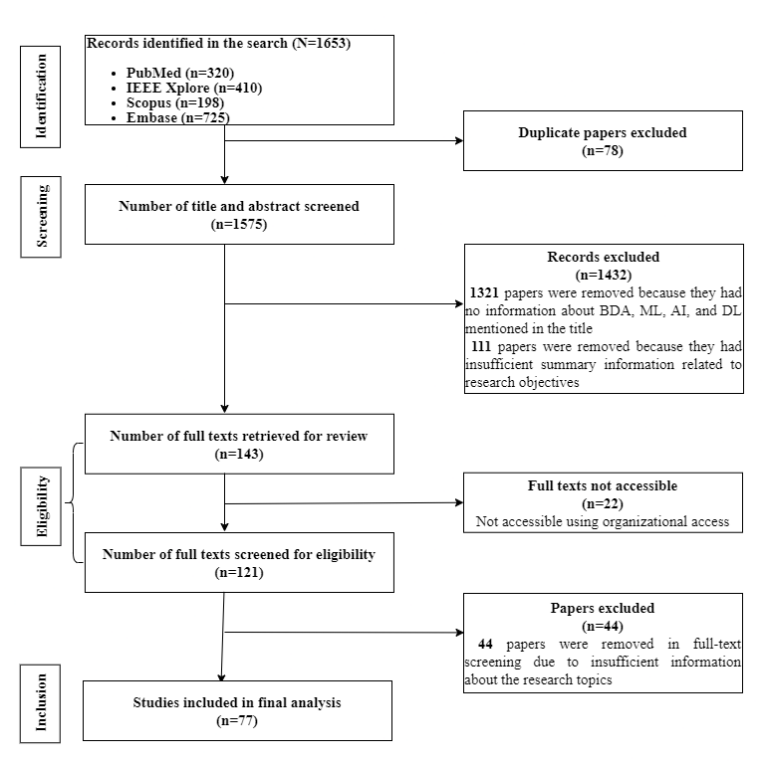
PRISMA (Preferred Reporting Items for Systematic Reviews and Meta-Analyses) flow diagram. AI: artificial intelligence; BDA: big data analytics; DL: deep learning; ML: machine learning.

Of the 1653 articles retrieved from the 4 databases, 78 (4.72%) duplicate publications were removed initially. Next, in the title and abstract screening stage, 83.87% (1321/1575) articles were removed as these did not align with the review objectives or were either workshop articles or not original studies (letters to editors and descriptions of keynotes). A total of 43.7% (111/254) of the publications were also excluded due to inadequate information related to research objectives. The remaining 56.3% (143/254) of the studies were taken for full-text review. Moreover, of the 143 studies, 22 (15.4%) were inaccessible and excluded accordingly. Finally, 121 (84.6%) of the 143 articles were reviewed in full text for eligibility. Subsequently, 44 (30.8%) were excluded due to inadequate information considering research objectives, and finally, 77 (53.8%) articles were selected for data extraction. A detailed description of the article selection process is given in the PRISMA (Preferred Reporting Items for Systematic Reviews and Meta-Analyses) flow diagram (Figure 1).

### Key Study Characteristics and Thematic Domains

This scoping review identified 77 research articles that examine how BDA, AI, ML, and DL are used in Bangladesh’s health care system and public health. The characteristics of the studies were grouped according to the study methodology, data types, and different health domains. Researchers mostly applied ML algorithms in their studies, and in 84% (65/77) of the studies, they used cross-sectional study data. The use of cohort study, case-control study, and retrospective study data was lower among researchers. Table 1 shows the characteristics of the selected studies according to different categories.

**Table 1:** Characteristics of included studies based on study design, types of data, and health domains (N=77).

Characteristics			Studies, n (%)
**Study design**			
	Cross-sectional study	65 (84)	
	Cohort study	5 (6)	
	Case-control study	4 (5)	
	Retrospective study	3 (4)	
**Type of data**			
	Primary	40 (52)	
	Secondary	37 (48)	
**Health domain**			
	Infectious diseases	15 (19)	
	Noncommunicable diseases	23 (30)	
	Child health	11 (14)	
	Mental health	9 (12)	
	Preventive health	4 (5)	
	Maternal health	5 (6)	
	Vector-borne diseases	5 (6)	
	Others	5 (6)	
**Technology domain**			
	Machine learning	65 (84)	
	Deep learning	7 (9)	
	Artificial intelligence	4 (5)	
	Big data analytics	1 (1)	

**Figure 2:**
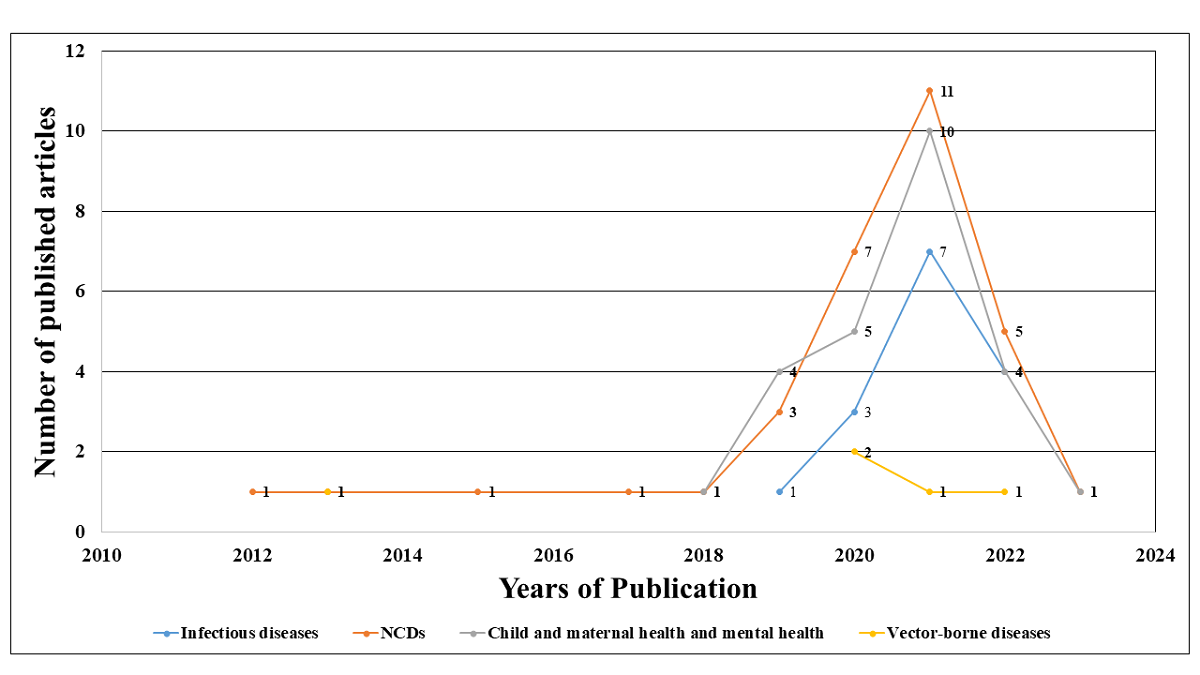
Number of articles on different health focuses by year, using big data analytics, artificial intelligence, machine learning, and deep learning. NCD: noncommunicable disease.

Although the date limiter was set from January 1, 2000, to September 10, 2023, no published articles according to the research objectives were found before 2012. A small number (4/77, 5%) of published research articles were found between 2012 and 2017, focusing on public health issues using BDA, AI, ML, and DL. After 2017, a substantial surge in interest among researchers in using AI, ML, and DL was observed, which mostly focused on NCDs, infectious diseases, and child health. Among the NCD group, predicting type 2 diabetes mellitus, hypertension, and cancer is mostly focused on, and among the infectious diseases, COVID-19 was mostly prevalent throughout the pandemic from 2020 to 2022. In addition, the researchers highlighted using the target technologies for different vector-borne diseases during this timeframe (Figure 2).

Table 2 summarizes the included articles with respect to their objectives, methods, dataset sizes, and investigated health issues.

**Table 2:** Summary of the selected studies.

Study	Health focus	Objective of the study	Outcomes
Rahman et al [17], 2021	Infectious diseases (COVID-19)	To categorize the patients with COVID-19 into 3 risk groups (low, moderate, and high) for predicting mortality risk among these patients	LR^a^ outperforms other models and its overall accuracy, weighted precision, sensitivity, specificity, and *F*_1_-score are 88%, 88%, 87%, and 90%, respectively.
Satu et al [18], 2021	Infectious diseases (COVID-19)	To develop a short-term forecasting model that predicts the severity of COVID-19	FPM^b^ showed better RMSE^c^, MAE^d^, and *R*² values for predicting COVID-19 cases.
Rafi [19], 2020	Infectious diseases (COVID-19)	To identify COVID-19 cases using CXR^e^ and classify them as patients who are normal or patients with COVID-19	Ensemble deep transfer learning model to identify COVID-19 cases with respect to normal cases, with an accuracy of 98.43%.
Sarkar et al [20], 2020	Infectious diseases (COVID-19)	To find the best algorithm that will predict COVID-19 cases, death cases, and recovery cases and the spreading pattern	FPM has a better RMSE value (33,785.55) than other models.
Mohammad Masum et al [21], 2020	Infectious diseases (COVID-19)	To forecast confirmed COVID-19 cases, deaths, and recovery	The LSTM^f^ model has an accurate RMSE value than that of SVR^g^ and RF^h^ on train and test validation sets.
Hassan et al [22], 2021	Infectious diseases (COVID-19)	To create a distributed AI^i^ on the edge that is able to deal with big data and to predict the course of the COVID-19 pandemic accurately.	DNN^j^ achieved a higher *R*^2^ value of 0.9501 and a better MAPE^k^ value of 13.789 in predicting the course of the outbreak.
Chowdhury et al [23], 2021	Infectious diseases (COVID-19)	To predict COVID-19 daily new cases with higher accuracy	The LSTM model performs better than the ANFIS^l^ model in predicting COVID-19 cases, with MAPE of 4.51, RMSE of 6.55, and correlation coefficient of 0.75.
Ullah et al [24], 2022	Infectious diseases (COVID-19)	To create AI-based diagnostic tool to detect COVID-19 accurately by examining CXR	At the testing stage, the designed model could interpret CXRs with a precision of 0.98, recall and sensitivity of 0.97, and *F*_1_-score of 67 0.97 for COVID-19. At the validation stage, it showed high sensitivity (90%) and specificity (92%) in detecting COVID-19. The AUC^m^ values for COVID-19 and pneumonia were 0.91 and 0.87, respectively.
Leon et al [25], 2021	Infectious diseases (COVID-19)	To predict the number of people infected with COVID-19 and the number of deaths for the upcoming month due to the COVID-19 pandemic.	FPM performs well in predicting the current affected cases with an RMSE value of 318.538, and the ARIMA^n^ model performs well in predicting death with an RMSE value of 10.708.
Rahman et al [26], 2019	Infectious diseases (COVID-19)	To predict COVID-19 cases in Bangladesh (daily newly infected cases, daily new fatality, and daily new recovered patients in the next 30 days range)	For daily infected cases forecasting, multiple linear regression and ridge regression perform best and obtain *R*^2^, MSE^o^, MAE, and RMSE values of 0.99, 0.004, 0.05, and 0.06, respectively. For daily new fatality cases forecasting, multiple linear regression, ridge regression, and Lasso regression performed best and obtained *R*^2^, MSE, MAE, and RMSE values of 0.99, 0.003, 0.05, and 0.06, respectively. For daily new recovery forecasting, multiple linear regression performed best and obtained *R*^2^, MSE, MAE, and RMSE values of 0.94, 0.05, 0.16, and 0.23, respectively. Overall, multiple linear regression outperforms all other models.
Haq et al [27], 2022	Infectious diseases (COVID-19)	To predict the future direction and spreading pattern of COVID-19	FPM is appropriate for forecasting the COVID-19 pandemic trend in Bangladesh.
Absar et al [28], 2020	Infectious diseases (COVID-19)	To predict the progression of COVID-19 for a period of more than a year under various scenarios in Bangladesh	LSTM showed better RMSE, MAE, and *R*² values for predicting COVID-19 cases.
Karmokar et al [29], 2022	Infectious diseases (COVID-19)	To analyze the effects of meteorological parameters on COVID-19	Cloudy weather has a positive association with COVID-19, temperature has a positive association with number of daily deaths and those who recovered, wind speed has a negative association with COVID-19, air quality has a negative association with daily deaths and recovered, air pressure has a weak negative relation with daily cases and deaths, and rain has a negative relation with daily cases.
Qin et al [30], 2021	Infectious diseases (tuberculosis)	To detect tuberculosis using AI algorithm on CXR images for triaging tuberculosis	Model could identify *Vibrio cholerae* O1 incidence with AUC 93.4%; 95% CI 92.1%-94.7%.
Azman et al [31], 2019	Infectious diseases (cholera)	To identify individuals infected with *V cholerae* O1	All the AI algorithms can be highly accurate and useful triage tools for tuberculosis detection in high-burden regions and outperform human readers with an accuracy of 90.81% (95% CI 90.33-91.29) for qXR, 90.34% (95% CI 89.81-90.87) for CAD4TB, 88.61% (95% CI 88.03-89.20) for Lunit INSIGHT CXR, 84.90% (95% CI 84.27-85.54) for InferRead DR, and 84.89% (95% CI 84.26-85.53) for JF CXR-1. Only qXR (specificity 74.3%; 95% CI 73.3-74.9) and CAD4TB (specificity 72.9%; 95% CI 72.3-73.5) met the TPP at 90% sensitivity.
Pranto et al [32], 2020	NCDs^p^ (diabetes)	To predict diabetes among female patients in Bangladesh	RF models showed better performance in predicting the patients with diabetes with an accuracy, *F*_1_-score, and AUC of 78%, 0.84, and 0.83, respectively, in the test set, and KNN^q^ showed better accuracy and *F*_1_-score (81.2% and 0.88) and naïve Bayes in AUC (0.84) in validation.
Nishat et al [33], 2021	NCDs (diabetes)	To predict diabetes mellitus	GP^r^ emerged as the best-performing algorithm, which is proposed as the most efficient classifier with accuracy, *F*_1_-score, and AUROC^s^ of 98.25%, 0.972, and 0.979, respectively.
Islam et al [34], 2020	NCDs (diabetes)	To automatically detect diabetes using ML^t^-based classifiers	Area of living, electricity, wealth index, age, education, working status, smoking status, medicine intake, weight, and BMI are significantly associated with diabetes at a 5% significance level. Furthermore, bagged CART^u^ provides the highest accuracy and AUC of 94.3% and 0.600.
Jahan et al [35], 2020	NCDs (diabetes)	To identify patients with a high risk of diabetes using ML	IBK^v^ performs well in identifying patients with a high risk of diabetes with 98.73% accuracy.
Emon et al [36], 2021	NCDs (diabetes)	To predict diabetes at an early stage by identifying symptoms and disease related to diabetes	RF performs well in identifying patients with a high risk of diabetes with an accuracy of 98%.
Dutta et al [37], 2022	NCDs (diabetes)	To prepare a diabetes disease classifier dataset so that patients with high risk of diabetes could be identified early	Ensemble-based model (DT^w^, RF, XGB^x^, and LGBM^y^) proclaims an accuracy of 73.5% and an AUC of 83.2% in predicting diabetes.
Wood [38], 2022	NCDs (diabetes)	To identify the signs and symptoms of type 2 diabetes and predicted type 2 diabetes using ML and statistical method	Support vector classifier predicts early‐onset type 2 diabetes with 2.1% errors.
Asaduzzaman et al [39], 2021	NCDs (cancer)	To identify factors that have a greater influence on the severity of cervical cancer and ovarian cancer	AdaBoost performed the best with a classification accuracy of 78.8% in orange and 79% in Sklearn for neurodegenerative disease. In the case of cervical cancer, LR provides the best score of 84.8%, and with Sklearn, the score was 79.3%. On the other hand, SVM^z^ shows the best accuracy of 88.3% in orange, and the DT provides 98.6% classification accuracy in Sklearn for ovarian cancer.
Maliha et al [40], 2019	NCDs (cancer)	To predict cancer disease and identified the factors associated with cancer disease	The KNN algorithm achieved a higher accuracy in predicting cancer disease with an accuracy of 98.8%.
Rejaul Islam Royel et al [41], 2021	NCDs (cancer)	To design a tool for early detection of stomach cancer risk level	The study identified 18 significant top risk factors of stomach cancer.
Ahmed et al [42], 2013	NCDs (cancer)	To predict patients with a risk of lung cancer	AprioriTid and DT algorithms can be used to identify the frequent patterns.
Rashid et al [43], 2022	NCDs (diabetes)	To investigate the use of ML approach for predicting cardiac autonomic neuropathy, diabetic peripheral neuropathy, and diabetic retinopathy using only the patient demographic, clinical, and laboratory profiles	RF performs well using diastolic blood pressure, albumin-creatinine ratio, and gender for CAN^aa^ testing (98.67%); microalbuminuria, smoking history, and hemoglobin A1c for DPN^ab^ testing (67.78%); and hemoglobin A1c, microalbuminuria, and smoking history for RET^ac^ testing (84.38%).
Kayyum et al [44], 2020	NCDs (CVD^ad^)	To predict the chances of myocardial infarction occurring so that people can take precautions and take measures to prevent it	Bagging can predict patients who are at a high risk of myocardial infarctions with an accuracy of 93.913%, *F*_1_-score of 0.940, and ROC of 0.974.
Tabassum et al [45], 2019	NCDs (CVD)	To predict the cardiac status of a patient or of a person who is unaware of his or her cardiac condition	Neural network showed 82% accuracy.
Chowdhury et al. [46], 2021	NCDs (CVD)	To predict heart disease by analyzing important features from health assessment	SVM has better accuracy in predicting heart disease with an accuracy of 91%.
Fahim et al [47], 2022	NCDs (CVD)	To detect patients with a high risk of CVD at an early stage	XGB performs well in identifying high-risk patients accurately, with an accuracy of 73.72%. Furthermore, the model showed 81.14% accuracy in the dataset, including smoking and alcohol intake.
Islam et al [48], 2021	NCDs (hypertension)	To characterize the risk factors of hypertension among adults in Bangladesh	The combination of SVMRFE-GB^ae^ gives the maximum accuracy (66.98%), recall (97.92%), *F*_1_-score (78.99%), and AUC (0.669) compared to others in predicting the risk of hypertension among adults in Bangladesh.
Arefa et al [49], 2019	NCDs (hypertension)	To develop an ML model for a decision support system to predict early hypertension risk among patients and assessed the performance of different ML algorithms	DT, LR, SVM, KNN, and bagged tree showed an accuracy of 100%. Fine Gaussian SVM showed an accuracy of 94.6%. Coarse Gaussian SVM showed an accuracy of 86%.
Islam et al [50], 2022	NCDs (hypertension)	To predict hypertension and its associated factors and compared ML model’s performances	XGB, GBM^af^, LR, and LDA^ag^ models can predict hypertension with a greater accuracy of 90%; DT achieved a precision value of 91%; XGB, GBM, LR, and LDA achieved a recall value of 100%; and XGB, GBM, LR, and LDA scored *F*_1_-score of 95%.
Asadullah et al [51], 2023	NCDs (hypertension)	To predict patients with a high risk of hypertension	Proposed ensemble model showed a higher accuracy, *F*_1_-score, and AUC of 78.17%, 08751, and 0.8634, respectively, in predicting patients with a high risk of hypertension.
Ifraz et al [52], 2021	NCDs (CKD^ah^)	To provide a methodology for predicting CKD status using clinical data	The LR model performs better than other models to predict CKD with an accuracy of 97%.
Ehsan et al [53], 2021	NCDs (Parkinson disease)	To screen Parkinson disease using smartphone-based triaxial accelerometer data and cloud-based automated tool	RF classifier produced the optimum result with 88.33% accuracy, 90% sensitivity, and 85% specificity to effectively discriminate between patients with Parkinson disease and healthy individuals.
Akter Hossain et al [54], 2020	NCDs (leukemia disease)	To detect the WBC^ai^ components from whole blood images and counted them to know about the possibility of leukemia	Faster-RCNN^aj^ model could identify leukemia-positive properties with 90% accuracy.
Shahriar et al [55], 2019	Child health (malnutrition)	To identify the risk factors that had a strong association with malnutrition among children aged 0 to 59 months , predicting malnourished children	ANN^ak^ algorithm can predict risk factors better than other algorithm with an accuracy of 86%, 70%, and 67.3%, respectively, for wasting, underweight, and stunting child respectively.
Methun et al [56], 2021	Child health (morbidity)	To determine the factors that regulated the incidence of preventable outbreaks of disease or symptoms among children aged <5 years in Bangladesh	Logistic classifier identified the factors that regulate the incidence of preventable outbreaks of disease or symptoms among children aged <5 years in Bangladesh with an accuracy, *F*_1_-score, and AUC of 0.7, 0.812, and 0.621, respectively.
Borson et al [57], 2020	Child health (low birth weight)	To construct a predictive model for low birth weight prediction by analyzing health and demographic data related to neonatal health conditions in the context of Bangladesh	LR and SVM gained accuracy, precision, and recall scores of 80.30%, 0.743, and 0.803 and 80.29%, 0.803, and 0.803, respectively, in 10-fold cross validation and 81.66%, 0.817, and 0.817 and 81.67%, 0.817, and 0.817, respectively, in training and testing stage.
Rahman et al [58], 2021	Child health (malnutrition)	To predict malnourished children aged <5 years based on their risk factors	RF algorithm can classify stunted growth, wasted growth and underweight children accurately and obtained the highest accuracy of 88.3% for children with stunted growth, 87.7% for children with wasted growth, and 85.7% for children who are underweight.
Talukder and Ahammed [59], 2020	Child health (malnutrition)	To predict malnutrition status among children aged <5 years in Bangladesh	RF accurately identified malnutrition status among children aged <5 years, with an accuracy of 68.51%, a sensitivity of 94.66%, and a specificity of 69.76%.
Khan et al [60], 2021	Child health (malnutrition)	To identify risk factors of stunting among children aged <5 years in Bangladesh	Gradient boosting algorithm predicted stunted children with an accuracy of 67.47%.
Jehan et al [61], 2020	Child health (PTB^al^)	To investigate the ability of transcriptomics and proteomics profiling of plasma and metabolomics analysis of urine to identify early biological measurements associated with PTB	PTBs can be predicted using blood and urine samples collected early in the pregnancy, providing opportunities for interventions and the proposed model could be able to identify PTB with an AUROC of 0.83 (95% CI 0.72-0.91).
Islam Pollob et al [62], 2022	Child health (low birth weight)	To determine the risk factors of low birth weight and predicted babies with low birth weight based on ML algorithms	LR-based classifier provided the most accurate classification of babies with low birth weight and has 87.6% accuracy and 0.59 AUC.
Mehedi Hasan et al [63], 2021	Child health (pneumonia)	To classify and predict pneumonia positive and pneumonia negative cases using ML algorithm	DT model can predict pneumonia with high accuracy.
Aftab et al [64], 2021	Child health (GA^am^)	To develop and validate programmatically feasible and accurate approaches to estimate newborn GA in low-resource settings	The ML model (10 neonatal characteristics and LMP^a^^n^) estimated GA with a variability of 15.7 days of early ultrasound dating.
Mansur et al [65], 2021	Child health (malnutrition)	To study interactions among various sociodemographic risk factors of childhood stunting in Bangladesh	LR identified malnourished children with an accuracy of 69.4%.
Khudri et al [66], 2023	Maternal health (malnutrition)	To predict the BMI and the risks of malnutrition outcomes for Bangladeshi women of childbearing age from their economic, health, and demographic features	SVM and KNN are the 2 best-performing methods in BMI prediction, with *R*^2^ value of 23.9% and 23.2%, RMSE of 3.372 and 3.387, and MAE of 2.718 and 2.756, respectively.
Islam et al [67], 2022	Maternal health (malnutrition)	To identify potential risk factors of malnourished women and predicted malnourished women using ML algorithm	RF-based classifier provides 81.4% accuracy and 0.837 AUC for underweight prediction and 82.4% accuracy and 0.853 AUC for overweight or obese prediction.
Choudhury et al [68], 2019	Mental health	To identify depression among university students in an early stage using ML	RF algorithm accurately predicted depression successfully, with accuracy and *F*_1_-score of 75% and 60%, respectively.
Haque et al [69], 2021	Mental health	To understand milestone achievement of ASD^ao^ using mCARE mobile app and association of milestone achievement with family or child sociodemographic characteristics	For milestone for brushing teeth, LR, KNN, and ANN achieved 95% accuracy; for toilet use, 84% accuracy was achieved with KNN and ANN; and for urinates in toilet or potty and buttons large buttons, ANN achieved an accuracy of 91% and 76%, respectively. For all the parameters, ANN had a higher accuracy (approximately >80% on average).
Tariq et al [70], 2019	Mental health	To classify autism spectrum disorder, neurotypical disorder, and speech and language condition using ML classifier.	The proposed model achieved an accuracy (AUC) of 76% (SD 3%) and sensitivity of 76% (SD 4%) in identifying atypical children from among those with developmental delays. Additionally, the model demonstrated an accuracy (AUC) of 85% (SD 5%) and a sensitivity of 76% (SD 6%) for identifying children with ASD from those predicted to have other developmental delays.
Imtinan Uddin et al [71], 2020	Mental health	To predict early depression risk among technology employees in Bangladesh as well as found some key factors that contributed to depression	AdaBoost DT achieved approximately 98% accuracy in predicting employees with depression.
Khan et al [72], 2018	Mental health	To examine classification algorithm to predict mental disorder	RF produced a better result with an accuracy of 85.1% in predicting mental disorders in real datasets. Furthermore, in an enhanced dataset with synthesized data, it showed 85.9% accuracy.
Rois et al [73], 2021	Mental health	To detect significant risk factors of perceived stress and predicted the prevalence of stress among Bangladeshi university students using ML algorithm	RF model performed better and authentically predicted stress compared with other ML techniques with an accuracy of 0.8972, precision of 0.9241, sensitivity of 0.9250, specificity of 0.8148, AUROC of 0.8715, and k-fold accuracy of 0.8983.
Ahmed et al [74], 2020	Mental health	To detect depression and anxiety using ML	CNN^ap^ algorithm has the highest accuracy of 96% for anxiety and 96.8% for depression. In addition, the analysis showed that among Bangladeshi women aged 18 to 35 years, 7.4% experienced profound levels of anxiety and 15.6% experienced chronic depression.
Khan et al [75], 2018	Mental health	To examine the performance of classification algorithms to predict mental disorder	RF has a better performance than the other algorithms.
Moon et al [76], 2022	Mental health	To predict the mental development condition of rural children during the COVID-19 pandemic	RF achieved a higher accuracy of 92.41% in predicting individuals who are mentally hampered
Hu et al [77], 2017	Decision support system	To transform the prediction model into a field-deployable application to predict drug functions to support clinical decisions	GBDT^aq^ models could predict drugs with an accuracy of 96.2%.
Baba et al [78], 2015	Decision support system	To recommend high-cost and low-cost diagnostic tests, recommended drugs recommended for patients, and predicted future health-risk levels by assessing patients’ health status data	The approach with multiple classifiers successfully reduced the costs of health checkups, a multitask learning method provided accurate recommendations for specific types of drugs, and an active learning method achieved an efficient assignment of health care workers to the follow-up cares of patients.
Al Iqbal [79], 2012	Decision support system	A clinical decision support system to serve the rural medicine center	ACM-FOCL^ar^ can identify hepatitis and breast cancer with 90.17% and 84.97 accuracy, respectively, and FOCL^as^ can identify lung cancer, diabetes, heart disease, and arrhythmia with 82.67%, 81.60%, 86.88%, and 70.73%, respectively.
Bin Alam et al [80], 2021	Pregnancy and childbirth	To evaluate bagging classifiers in birth mode prediction and identified key factors influencing cesarean section	DT bagging produces the highest accuracy of 0.87 with a 0.60 *F*_1_-score for predicting birth mode.
Kowsher et al [81], 2021	Pregnancy and childbirth	To predict the mode of childbirth that will be appropriate for pregnant women	Quadratic discriminant analysis has the highest accuracy and *F*_1_-score of 0.97992 and 0.979960, respectively, in predicting the mode of childbirth.
Hossain et al [82], 2022	Pregnancy and childbirth	To predict unwanted pregnancies among married women in Bangladesh using ML	Elastic net regression algorithm showed the best results and the most accurate classification for predicting unwanted pregnancy among Bangladeshi women with an accuracy of 77.51%.
Sarma et al [83], 2020	Vector-borne diseases (malaria)	To predict dengue fever at an early stage using ML algorithm	DT algorithm performs well in classifying normal fever and dengue fever, with an accuracy of 79%.
Dey et al [84], 2022	Vector-borne diseases (malaria)	To develop an ML model that can use relevant information about the factors that cause dengue outbreaks within a geographic region	SVR identified the dengue outbreak accurately with a prediction accuracy of 75%.
Islam et al [85], 2021	Vector-borne diseases (malaria)	To predict the probability of dengue fever before taking the pathological test	DT and naïve Bayes achieved a higher accuracy of 100% in predicting dengue fever.
Kassim et al [86], 2021	Vector-borne diseases (malaria)	To detect RBCs^at^ in malaria diagnostic smears using dual deep learning	The proposed RBCNet^au^ architecture detected RBC with *F*_1_-score, precision, and recall of 97.76%, 97.51%, and 98.07%, respectively.
Vink et al [87], 2013	Vector-borne diseases (malaria)	To detect malaria parasite using vision-based malaria parasite selection	In healthy samples, the system achieved an overall specificity of 99.999978% at the level of (infected) RBCs and a sensitivity of 75% at the cell level, enabling the detection of low parasite densities in a fast and easy-to-use manner.
Masud et al [88], 2020	Preventive health (appendicitis)	To identify risk factors for appendicitis using preoperative symptoms and developed an Android-based app	Apriori algorithm could correctly classify affected patients between medium to very high risk with 99% accuracy.
Sampa et al [89], 2020	Preventive health (uric acid)	To predict the blood uric acid level using ML model based on basic health checkup test results, dietary information, and sociodemographic information	Boosted DT regression can predict patients with a high risk of increased uric acid levels with an RMSE of 0.03.
Ferdowsy et al [90], 2021	Preventive health (obesity)	To predict obesity by identifying risk factors of obesity using ML-based data analysis	The LR algorithm performed better than other algorithms to predict obesity with an accuracy of 97.09%, whereas the gradient boosting algorithm produced the poorest accuracy of 64.08%.
Riajuliislam et al. [91], 2021	Preventive health (thyroid)	To predict hypothyroidism at an early stage by analyzing clinical and demographic data	RFE^av^ performed well in predicting features with an accuracy of 99.35%.
Diptu et al [92], 2018	Ophthalmology	To detect glaucoma with the data obtained from OCT^aw^ and tonometry test	ANFIS can detect glaucoma from OCT and tonometry tests with an accuracy of 81.25%.
Hassan et al [11], 2021	Health policy	To assess the policy and regulations for medical devices and policy for health data in Bangladesh and compared these with those in the United States and European Union. In addition, identified the gaps in implementing big data and ML in health care	The current regulatory framework of Bangladesh for medical device regulation is inadequate to regulate AI-based medical devices. Furthermore, Bangladesh has neither comprehensive data protection legislation nor specific sectoral laws for health data privacy.

^a^LR: logistic regression.

^b^FPM: Facebook Prophet model.

^c^RMSE: root mean square error.

^d^MAE: mean absolute error.

^e^CXR: chest x-ray.

^f^LSTM: long short-term memory.

^g^SVR: support vector regression.

^h^RF: random forest.

^i^AI: artificial intelligence.

^j^DNN: deep neural network.

^k^MAPE: mean absolute percentage error.

^l^ANFIS: adaptive neuro-fuzzy interference system.

^m^AUC: area under the curve.

^n^ARIMA: autoregressive integrated moving average.

^o^MSE: mean squared error.

^p^NCD: noncommunicable disease.

^q^KNN: k-nearest neighbor.

^r^GP: Gaussian process.

^s^AUROC: area under the receiver operating characteristic curve.

^t^ML: machine learning.

^u^CART: classification and regression tree.

^v^IBK: Instance-Based K-nearest Neighbors.

^w^DT: decision tree.

^x^XGB: extreme gradient boost.

^y^LGBM: light gradient boost Machine.

^z^SVM: support vector machine.

^aa^CAN: Convolutional Attention Network.

^ab^DPN: Dual Path Networks.

^ac^RET: RetinaNet.

^ad^CVD: cardiovascular disease.

^ae^SVMRFE-GB: Support Vector Machine Recursive Feature Elimination with Gradient Boosting.

^af^GBM: gradient boosting machine.

^ag^LDA: linear discriminant analysis.

^ah^CKD: chronic kidney disease.

^ai^WBC: white blood cell.

^aj^RCNN: Region-Based Convolutional Neural Networks.

^ak^ANN: artificial neural network.

^al^PTB: preterm birth.

^am^GA: gestational age.

^an^LMP: last menstrual period.

^ao^ASD: autism spectrum disorder.

^ap^CNN: convolutional neural network.

^aq^GBDT: gradient boosting decision tree.

^ar^ACN-FOCL: Attention Convolutional Network-Focused Clustering.

^as^FOCL: Focused Clustering.

^at^RBC: red blood cell.

^au^RBCNet: Residual Bi-directional Convolutional Network.

^av^REF: Recursive Elimination Feature.

^aw^OCT: optical coherence tomography.

### Synthesis of Findings

According to the findings, the studies intended to predict various diseases using various risk factor data (33/77, 43%), classify patients in disease-exposed and unexposed groups (21/77, 27%), predict trends of the outbreak (18/77, 23%), formulate intelligent systems for health service (3/77, 4%), and so on. One study explored the regulatory challenges of using big data technology in the health sector, as well as making recommendations for future policy. Another study tried to detect glaucoma with the data obtained from optical coherence tomography and the tonometry test.

The study’s findings indicate that, compared to secondary data, the use of prediction and classification algorithms on primary data has significantly grown (40/77, 52%). In a significant number of studies (37/77, 48%), researchers used data from the Bangladesh Demographic and Health Survey, which is part of the global Demographic and Health Surveys program, designed to collect information on fertility, family planning, and maternal and child health. Furthermore, a substantial portion of studies (12/77, 16%) made use of publicly available data from web-based sources, such as Kaggle, the University of California, Irvine repository, and Worldometer. Researchers validated their prediction algorithms within the context of Bangladesh.

In this review, we observed the adoption of digital interventions, including the use of mobile devices for public health purposes. This review explored articles (4/77, 5%) that leveraged mobile devices for their research, creating intelligent risk prediction tools by analyzing data collected through ML- and DL-based algorithms. The objective was to demonstrate the value of mobile apps in obtaining real-time health data and predicting health status. The primary outcomes of these studies underscored the utility of intelligent mobile apps for self-assessing an individual’s health and offering essential therapy recommendations.

On the basis of the data extracted in this review, 44 distinct AI, ML, and DL algorithms commonly used in public health studies were identified. Table 3 presents the algorithms according to different categories and their respective use frequencies.

**Table 3:** Different categories of available algorithms found in this scoping review (N=77).

Category and subcategory			Algorithm		Frequency, n (%)
**Supervised**					
	Classification	Decision tree		37 (48)	
	Classification	Random forest		37 (48)	
	Classification	Logistic regression		35 (45)	
	Classification	k-nearest neighbor		27 (35)	
	Classification	Naive Bayes		22 (29)	
	Classification	Quadratic discriminant analysis		1 (1)	
	Classification	Bagging classifier		1 (1)	
	Classification	Bagged classification and regression tree		1 (1)	
	Classification	CatBoost		1 (1)	
	Classification or regression	Artificial neural network		11 (14)	
	Classification or regression	Convolutional neural network		7 (9)	
	Regression	Gradient boosting		9 (12)	
	Regression	Linear regression		8 (10)	
	Regression	Adaptive boosting		7 (9)	
	Regression	Support vector regression		2 (3)	
	Regression	Elastic net regression		2 (3)	
	Regression	Gaussian process		1 (1)	
	Regression	Ridge regression		1 (1)	
	Regression	Multiple linear regression		1 (1)	
	Regression	Least absolute shrinkage and selection operator		4 (5)	
	Regression	Autoregressive		3 (4)	
	Time series	Facebook prophet time series model		5 (6)	
	Time series	Recurrent neural network		2 (3)	
	Time series	Long short-term memory		2 (3)	
**Unsupervised**					
	Clustering	k-means clustering		1 (1)	
	Dimensionality reduction	Principal component analysis		1 (1)	
	Time series	Autoregressive integrated moving average		4 (5)	
	Time series	Apriori algorithm		2 (3)	

Among these, the random forest algorithm emerged as a standout performer, delivering the highest accuracy in 21% (16/77) of the studies. The decision tree classifier was also highly effective, with a track record of accurate predictions in 16% (12/77) of the studies. However, for maternal and child health, logistic regression showed substantial promises, while for outbreak trend prediction, the Facebook Prophet model and long short-term memory (LSTM) demonstrated strong performance. In the mental health domain, random forest excelled, and for NCDs, random forest and support vector machine exhibited strong predictive capabilities. In the context of vector-borne diseases, the decision tree algorithm proved effective.

Several other algorithms showcased their utility in various domains, including support vector machine (9/77, 12%), logistic regression (8/77, 10%), Naïve Bayes (4/77, 5%), k-nearest neighbor (3/77, 4%), convolutional neural network (3/77, 4%), gradient boosting (3/77, 4%), LSTM (3/77, 4%), Facebook Prophet time series model (3/77, 4%), artificial neural network (2/77, 3%), XGBoost (2/77, 3%), a priori algorithm (2/77, 3%), and linear discriminant analysis (1/77, 1%). In addition, support vector regression (1/77, 1%), bagging classifier (1/77, 1%), quadratic discriminant analysis (1/77, 1%), multiple linear regression (1/77, 1%), elastic net regression (1/77, 1%), and bagged classification and regression tree (1/77, 1%) displayed their potential in achieving high prediction accuracy in their respective research studies.

This comprehensive analysis highlights the diverse and effective use of these algorithms in public health research, offering valuable insights for researchers and professionals in the field.

### Practical Implication of Studies

This review identified a number (9/77, 12%) [17,24,39,42,47,50,54,87,88] of studies where researchers have made web-based and Android-based solutions by applying AI-, ML- and DL-based models after conducting their research. Of the 9 studies, 6 (67%) studies developed apps that mostly focused on NCDs to allow persons to self-assess their own physical condition. The other 3 (33%) studies developed web-based prediction tools for identifying the trends and hot spots of the COVID-19 pandemic, which were found effective when the world suffered a lot from the pandemic situation.

### Limitations Mentioned by the Studies

According to the findings, this scoping review observed a significant number of limitations mentioned by the researchers. In approximately 27% (21/77) studies, researchers have expressed their concerns about the datasets they have used. The articles also expressed concerns about using a large amount of data with different dimensions for risk factor identification and prediction of NCDs. Sociodemographic data (8/77, 10%), clinical feature data (15/77, 19%), and lifestyle data (12/77, 16%) were crucial for effective risk factor identification as well as disease prediction, as mentioned in these 21 studies. However, the researchers were able to work on a small sample size with inadequate features, which overfitted their predictions, as reported by the articles.

For outbreak prediction, in 25% (19/77) of the studies, researchers mentioned the necessity to use diversified data from different dimensions with proper tuning of the AI, ML, and DL models on a regular basis. Environmental data, weather data, population movement data, quarantine data, vaccination information, clinical symptoms of patients, as well as crowd-sourced data were required for effective outbreak prediction. However, due to the lack of these features, they expressed concern that their provided prediction model may not work perfectly in all contexts.

Data source is also vital for an effective predictive model, as mentioned in some of the studies (12/77, 16%). Primary-sourced datasets seemed suitable for predictive modeling as they are usually collected in accordance with the research objective, whereas secondary-sourced datasets have their own research objectives, which was not convenient for effective predictive modeling.

Risk factor identification and prediction modeling were revealed as very sensitive for child health–related diseases. Some researchers mentioned using factors from different domains as well as clinical features (10/77, 13%).

## Discussion

### Principal Findings

This scoping review is an attempt to explore research focusing on Bangladesh’s perspective on using BDA, AI, ML, and DL technologies across various health care domains. The 77 studies included in this review were mostly quantitative in nature and focused on 7 distinct health care domains, including infectious diseases, NCDs, maternal health, child health, preventive health, vector-borne diseases, and mental health. While most (65/77, 84%) studies adopted a cross-sectional study design, a few used cohort (5/77, 6%), case-control (4/77, 5%), and retrospective (3/77, 4%) designs. The review revealed 44 distinct AI, ML, and DL algorithms for risk factor identification, classification, prediction, and trend analysis. The most used algorithms were decision tree, random forest, logistic regression, Naïve Bayes, artificial neural network, and k-nearest neighbor. Notably, among the studies, the random forest surpassed others in classification and prediction by accuracy and *F*_1_-score, followed by decision trees. However, in trend analysis, the Facebook Prophet model and LSTM performed better.

These technologies have exhibited effectiveness in classification, prediction, and severity analysis across various health care domains. For instance, time series algorithms, such as Facebook Prophet model and LSTM, can be invaluable in potential outbreak prediction, such as the COVID-19 pandemic. Sarkar et al [20] explored better performance in forecasting and severity analysis of COVID-19 outbreaks in Bangladesh using the Facebook Prophet model. Similarly, these algorithms can be of great use in addressing other epidemics and endemics, such as dengue and diarrhea outbreaks in Bangladesh and other South Asian countries. The use of these algorithms may include but is not limited to hot spot detection, disease pattern recognition, disease severity detection, and resource allocation for effective management. Moreover, Islam et al [48] and Asaduzzaman et al [39] incorporated different ML techniques (decision tree, support vector machine, XGBoost, gradient boosting machine, logistic regression, and linear discriminant analysis) to identify individuals at risk of NCDs, such as diabetes, hypertension, and cancer, as well as explored potential factors influencing the severity, considering the growing burden of NCDs in Bangladesh.

Furthermore, ML-driven classification algorithms can play a pivotal role in categorizing patients based on disease severity, facilitating efficient resource allocation in health care facilities. For example, Rahman et al [17] developed a web application incorporating ML algorithms to identify the severity of COVID-19 among patients in Bangladesh, enabling health care providers to prioritize treatment and intervention strategies effectively. Such categorization technology can reduce human error and overall stress if incorporated into high mobility wards (eg, diarrhea and casualty) and emergency rooms. However, future studies that focus on efficient fine-tuning and validation strategies are needed to ensure higher specificity and prevent false alarms.

Apart from these, several clinical decision support systems have been developed to assist depression diagnosis, considering the recent conversations on mental health, anxiety, and depression [77,79]. Although the models were proven successful in predicting and classifying information to aid public health experts, their real-world validation remains somewhat limited. Few studies (0/77, 13%) tested algorithms in controlled clinical settings with smaller sample sizes [17,32,36,41,49,70,73,76,83,88,89], while others (9/77, 12%) developed AI-, ML-, and DL-based applications without any real-world effectiveness testing [17,24,39,42,47,50,54,87,88].

In Bangladesh, the application of AI, ML, and DL in health care and public health has primarily been confined to risk factors identification, classification of exposed and unexposed disease groups, early danger-sign prediction, trend analysis, and early forecasting of outbreaks on a smaller scale. However, studies from high-income countries have presented the successful implementation of AI, ML, and DL interventions in diverse health care settings as well as mentioned stronger policy evidence [93-96]. For example, scoping reviews mentioned the successful use of BDA for mental health treatment [97], decision support management [98], patient classification, emergency department triage, and hospital resource allocation [99] as well as effective management of virus transmissions such as influenza, Zika virus, AIDS, chikungunya, and Ebola [100]. In Bangladesh, except for 1 policy article on big data, no studies mentioned any call for policies or actions on BDA for the public health domain [11].

While AI and ML have been used to forecast potential outbreaks such as COVID-19, influenza, and dengue in Bangladesh, BDA could be used in such domains for more comprehensive outcomes. Moreover, despite remarkable performances in screening, diagnosis, and prediction of cardiovascular diseases, the use of BDA in clinical settings remains limited [101]. Nonetheless, AI and ML have the potential to improve public health efforts and promote quality health care for all individuals across diverse communities, as mentioned by 1 review article [102].

### Research Implications

Our scoping review unveiled that integrating ML algorithms for diagnosis improves accuracy, saving time and effort. Moreover, AI-based decision support systems enhance prescription recommendations, aiding health care professionals in resource-constrained settings such as Bangladesh. Although ML and AI algorithms showed potential regarding outbreak trend prediction and hot spot detection, most studies were conducted on a smaller scale with a smaller sample size and within controlled environments. To gain a better understanding of the effectiveness of AI, ML, and DL algorithm–based prediction models and decision support systems, it is imperative to conduct large-scale studies in diverse environments and real clinical settings. Furthermore, in countries with robust health care systems, ML-based prediction systems tend to be successful in analyzing patient data from electronic health records and generating illness scores for predicting future health care needs [6]. For example, the Cambridge Adjutorium system, developed by a team at the Cambridge Centre for AI in Medicine in the United Kingdom, uses cutting-edge ML techniques to accurately forecast death rates, the necessity for intensive care unit admission, and the need for ventilation in patients with COVID-19 [103]. AI-powered physicians can offer health advice for common medical conditions, reducing hospital visits and staying through digital health coaching. Yet, applying these approaches in lower-income countries such as Bangladesh is limited due to structural, regulatory, and legislative constraints. Further studies and future research are essential to assess the effectiveness of AI-, ML-, and DL-based solutions in genuine clinical and larger population-based settings.

### Policy Implications

Technology has become integral to Bangladesh’s health care system, with platforms such as District Health Information System 2, electronic management information system, and open medical record system collecting vast amounts of patient data [12]. Despite challenges in structured data acquisition, data security, and processing capacity, research and implementation of AI, ML, and DL initiatives are expanding, promising substantial benefits for resource-limited countries such as Bangladesh. The government of Bangladesh is actively integrating AI, ML, and knowledge-based systems into health care operations as part of its strategic plan [104]. They are supporting technology industries and small teams in developing such solutions. Moreover, the government of Bangladesh is hosting national and international events and offering small grants for AI-driven health care projects, reflecting a strong commitment to incorporating these technologies despite financial limitations.

Training health care workers and ensuring consistent data entry are crucial to enhancing data integration and interoperability. Implementing AI, ML, and DL pilot projects can help optimize resources and gather insights for incremental and broader implementation. Moreover, collaborating with private technology companies, universities, and international organizations through public-private partnerships can codevelop and fund these initiatives, easing the financial burden on the government.

Furthermore, it is essential to develop robust data privacy and security frameworks suited to local needs and establish standards for the ethical use of AI, ML, and DL technologies. Investing in training health care workers and IT professionals, as well as encouraging local universities to include AI and ML in their curricula, will build a skilled workforce. In addition, targeted funding should prioritize high-return investments such as predictive analytics and AI-assisted diagnostic tools supported by thorough cost-benefit analyses.

Despite budget constraints, these strategic initiatives can potentially promote AI- and ML-based solutions in health care settings. By focusing on these actionable steps, Bangladesh can effectively integrate these technologies, ensuring sustainable advancements in digital health and improved overall health outcomes.

### Strengths and Limitations

Our study’s primary strength lies in the rigorous adherence to the scoping review methodology at every stage. This encompassed thorough screening and quality assessment of the studies included in our analysis. To our knowledge, this is the first initiative to outline the extension and utility of AI, ML, DL, and BDA in health care, along with illustrating the potential scope for interventions, research, and policies around such burgeoning domains in Bangladesh, which is also applicable for similar resource-limited settings. However, further studies are needed to investigate and contrast findings from other settings.

Nonetheless, our study does have other limitations. First, we exclusively considered literature in the English language, potentially excluding studies in other languages that could have offered valuable insights into the applications of DL, AI, ML, and BDA. However, it is worth noting that previous research has suggested that language restrictions in scoping reviews do not substantially introduce bias [105]. Moreover, we only included MEDLINE, IEEE Xplore, Scopus, and Embase databases to search the relevant articles, whereas we acknowledge that there might be potential useful insights from gray literature, including government of Bangladesh reports and nongovernmental organization publications. However, our included databases thoroughly cover peer-reviewed literature in medicine, engineering, and public health, aligning closely with our scoping review’s subject matter and ensuring the reliability and credibility of the evidence synthesized. Furthermore, several studies in our analysis involved small population sizes, and the overall quality of many included studies left room for doubt. Consequently, caution is warranted when interpreting our findings. Another limitation pertained to including studies that relied on secondary datasets and global data sources, primarily due to the scarcity of datasets specific to Bangladesh. We excluded 22 (15.4%) of the 143 articles from our review due to inaccessibility, which may have contributed valuable insights to our review. Finally, some studies did not mention explicit information about their ML models. Because our primary objective did not involve assessing the quality of modeling, these studies were included as part of our analysis.

### Conclusions

This scoping review outlines available research on BDA, AI, ML, and DL in the health care system sector of Bangladesh. The increasing demand for these cross-cutting technologies for analyzing and processing health data is steadily expanding. The use of ML for disease prediction and classification can aid health care professionals in enhancing diagnosis and treatments. Advanced algorithmic scoring systems based on multiple health determinants have immense potential to predict diagnosis, readmission, and mortality rates in the near future, potentially saving resources and efforts. Therefore, it is crucial to use BDA, AI, ML, and DL technology in designing future health interventions and policies to maximize the use of health resources and maintain optimum competitiveness. Finally, it is crucial to identify priority areas for the application of these technologies and develop a national strategy for the integration of AI and data science. This strategy should include but not be limited to components such as data monitoring, privacy and security, data availability and validation for research, academic and industry collaboration, e-infrastructure establishment, fostering a supportive legal framework, and training data scientists to ensure a successful transition to a future digital health era.

## Appendix

Multimedia Appendix 1 PRISMA-ScR (Preferred Reporting Items for Systematic Reviews and Meta-Analyses Extension for Scoping Reviews) checklist.

Multimedia Appendix 2 Database search strategy.

Multimedia Appendix 3 Quality assessment.

## Abbreviations

AI: artificial intelligence
BDA: big data analytics
DL: deep learning
LSTM: long short-term memory
ML: machine learning
NCD: noncommunicable disease
PRISMA: Preferred Reporting Items for Systematic Reviews and Meta-Analyses
PRISMA-ScR: Preferred Reporting Items for Systematic Reviews and Meta-Analyses Extension for Scoping Reviews

## Data Availability

All data generated or analyzed during this study are included in this published article (and its supplementary information files).
