# Supplementary material for: Implications of Big Data Analytics, AI, Machine Learning, and Deep Learning in the Health Care System of Bangladesh: Scoping Review
Source: J Med Internet Res. 2024 Oct 28;26:e54710. doi: 10.2196/54710 (PMC11555453; doi:10.2196/54710)
Supplement: Multimedia Appendix 2 [file jmir_v26i1e54710_app2.pdf]

Search Summary-1

Name of the database: PubMed (Advanced)

Date of search: 10th September, 2023

Timeline: 1st January, 2000 to 10th September, 2023

Search Language

Line #	Theme	Search Language	Output #
1	Artificial Intelligence	"Artificial Intelligence"[MeSH Terms]	178258
2		Artificial Intelligence[Text Word]	61349
3		AI[Text Word]	54130
4		Machine Intelligence[Text Word]	299
5		Cognitive Computing[Text Word]	108
6		Intelligent System[Text Word]	558
7		Automated Reasoning[Text Word]	111
8		Computational Intelligence[Text Word]	502
9		Computer Reasoning[Text Word]	7
10		Computer Vision System*[Text Word]	351
12		(((((((("Artificial Intelligence"[MeSH Terms]) OR ("Artificial Intelligence"[Text Word])) OR ("AI"[Text Word])) OR ("Machine Intelligence"[Text Word])) OR ("Cognitive Computing"[Text Word])) OR ("Intelligent System"[Text Word])) OR ("Automated Reasoning"[Text Word])) OR ("Computational Intelligence"[Text Word])) OR ("Computer Reasoning"[Text Word])) OR ("Computer Vision System*"[Text Word])	235847
13	Machine Learning	("Mesh_Terms":"Machine Learning")	59692
14		Machine Learning[Text Word]	101308
15		Transfer Learning[Text Word]	5325
16		Statistical Learning[Text Word]	2325
17		Predictive modelling[Text Word]	981
18		Pattern Recognition[Text Word]	84493
19		Unsupervised Learning[Text Word]	2655
20		Supervised Learning[Text Word]	5610
21		Pattern Recognition System[Text Word]	230
22		Deep Neural Learning[Text Word]	16
23		((((((((("Machine Learning"[MeSH Terms]) OR ("Machine Learning"[Text Word])) OR	210912

		("Transfer Learning"[Text Word])) OR ("Statistical Learning"[Text Word])) OR ("Predictive modelling"[Text Word])) OR ("Pattern Recognition"[Text Word])) OR ("Unsupervised Learning"[Text Word])) OR ("Supervised Learning"[Text Word])) OR ("Pattern Recognition System"[Text Word])) OR ("Deep Neural Learning"[Text Word])	
24	Deep Learning	("Mesh_Terms":"Deep Learning")	16433
25		Deep Learning[Text Word]	53398
26		Neural Network*[Text Word]	110692
27		Artificial Neural Network*[Text Word]	17919
28		Deep Neural Network*[Text Word]	9520
29		Hierarchical Learning[Text Word]	106
30		((((("Deep Learning"[MeSH Terms]) OR ("Deep Learning"[Text Word])) OR ("Neural Network*"[Text Word])) OR ("Artificial Neural Network*"[Text Word])) OR ("Deep Neural Network*"[Text Word])) OR ("Hierarchical Learning"[Text Word])	138762
31	Big Data	("Mesh_Terms":"Big Data")	2857
32		Big Data[Text Word]	14658
33		Data Mining[Text Word]	21337
34		Big Data Analytics[Text Word]	872
35		Data Science[Text Word]	3483
36		Predictive Analytics[Text Word]	922
37		Data-driven Decision Making[Text Word]	247
38		Large-scale Data Analysis[Text Word]	175
39		((((((("Big Data"[MeSH Terms]) OR ("Big Data"[Text Word])) OR ("Data Mining"[Text Word])) OR ("Big Data Analytics"[Text Word])) OR ("Data Science"[Text Word])) OR ("Predictive Analytics"[Text Word])) OR ("Data-driven Decision Making"[Text Word])) OR ("Large-scale Data Analysis"[Text Word])	38679
40	Healthcare System	("Mesh_Terms":"Public Health")	9281158
41		Public Health[Text Word]	435923
42		Population Health[Text Word]	17829
43		Community Health[Text Word]	91824
44		Epidemiology[Text Word]	2240319

45		Healthcare[Text Word]	105981
46		Disease Prevention[Text Word]	62842
47		Medical Care[Text Word]	7938
48		Public Health Informatics[Text Word]	1505
49		"Public Health Surveillance "[MeSH Terms]	5237
50		"Public Health Informatics"[MeSH Terms]	1279
51		((((((((("Public Health"[MeSH Terms]) OR ("Public Health"[Text Word])) OR ("Population Health"[Text Word])) OR ("Community Health"[Text Word])) OR ("Epidemiology"[Text Word])) OR ("Healthcare"[Text Word])) OR ("Disease Prevention"[Text Word])) OR ("Medical Care"[Text Word])) OR ("Public Health Informatics"[Text Word])) OR ("Public Health Surveillance "[MeSH Terms])) OR ("Public Health Informatics"[MeSH Terms])	9963073
52	Bangladesh	("Mesh_Terms":"Bangladesh")	14499
53		Bangladesh[MeSH Terms]	21923
54		Bangladeshi[MeSH Terms]	3968
55		Bangladesh Health System[MeSH Terms]	8
56		"Bangladesh"[Affiliation]	28354
57		(((("Bangladesh"[MeSH Terms]) OR ("Bangladesh"[Text Word])) OR ("Bangladeshi"[Text Word])) OR ("Bangladesh Health System"[Text Word])) OR ("Bangladesh"[Affiliation])	38409
58		((((("Bangladesh"[MeSH Terms]) OR ("Bangladesh"[Text Word])) OR ("Bangladeshi"[Text Word])) OR ("Bangladesh Health System"[Text Word])) OR ("Bangladesh"[Affiliation])) AND ((((((((((("Public Health"[MeSH Terms]) OR ("Public Health"[Text Word])) OR ("Population Health"[Text Word])) OR ("Community Health"[Text Word])) OR ("Epidemiology"[Text Word])) OR ("Healthcare "[Text Word])) OR ("Disease Prevention"[Text Word])) OR ("Medical Care "[Text Word])) OR ("Public Health	19634

		Informatics"[Text Word])) OR ("Public Health Surveillance "[MeSH Terms])) OR ("Public Health Informatics"[MeSH Terms]))	
59		((((((((((("Artificial Intelligence"[MeSH Terms]) OR ("Artificial Intelligence"[Text Word])) OR ("AI"[Text Word])) OR ("Machine Intelligence"[Text Word])) OR ("Cognitive Computing"[Text Word])) OR ("Intelligent System"[Text Word])) OR ("Automated Reasoning"[Text Word])) OR ("Computational Intelligence"[Text Word])) OR ("Computer Reasoning"[Text Word])) OR ("Computer Vision System*"[Text Word])) OR (((((((((("Machine Learning"[MeSH Terms]) OR ("Machine Learning"[Text Word])) OR ("Transfer Learning"[Text Word])) OR ("Statistical Learning"[Text Word])) OR ("Predictive modelling"[Text Word])) OR ("Pattern Recognition"[Text Word])) OR ("Unsupervised Learning"[Text Word])) OR ("Supervised Learning"[Text Word])) OR ("Pattern Recognition System"[Text Word])) OR ("Deep Neural Learning"[Text Word]))) OR (((((("Deep Learning"[MeSH Terms]) OR ("Deep Learning"[Text Word])) OR ("Neural Network*"[Text Word])) OR ("Artificial Neural Network*"[Text Word])) OR ("Deep Neural Network*"[Text Word])) OR ("Hierarchical Learning"[Text Word]))) OR (((((((("Big Data"[MeSH Terms]) OR ("Big Data"[Text Word])) OR ("Data Mining"[Text Word])) OR ("Big Data Analytics"[Text Word])) OR ("Data Science"[Text Word])) OR ("Predictive Analytics"[Text Word])) OR ("Data-driven Decision Making"[Text Word])) OR ("Large-scale Data Analysis"[Text Word]))	438694
60		(((((((((((("Artificial Intelligence"[MeSH Terms]) OR ("Artificial Intelligence"[Text Word])) OR ("AI"[Text Word])) OR	320

		("Machine Intelligence"[Text Word])) OR ("Cognitive Computing"[Text Word])) OR ("Intelligent System"[Text Word])) OR ("Automated Reasoning"[Text Word])) OR ("Computational Intelligence"[Text Word])) OR ("Computer Reasoning"[Text Word])) OR ("Computer Vision System*"[Text Word])) OR (((((((((("Machine Learning"[MeSH Terms]) OR ("Machine Learning"[Text Word])) OR ("Transfer Learning"[Text Word])) OR ("Statistical Learning"[Text Word])) OR ("Predictive modelling"[Text Word])) OR ("Pattern Recognition"[Text Word])) OR ("Unsupervised Learning"[Text Word])) OR ("Supervised Learning"[Text Word])) OR ("Pattern Recognition System"[Text Word])) OR ("Deep Neural Learning"[Text Word]))) OR (((((("Deep Learning"[MeSH Terms]) OR ("Deep Learning"[Text Word])) OR ("Neural Network*"[Text Word])) OR ("Artificial Neural Network*"[Text Word])) OR ("Deep Neural Network*"[Text Word])) OR ("Hierarchical Learning"[Text Word]))) OR (((((((("Big Data"[MeSH Terms]) OR ("Big Data"[Text Word])) OR ("Data Mining"[Text Word])) OR ("Big Data Analytics"[Text Word])) OR ("Data Science"[Text Word])) OR ("Predictive Analytics"[Text Word])) OR ("Data-driven Decision Making"[Text Word])) OR ("Large-scale Data Analysis"[Text Word]))) AND (((((("Bangladesh"[MeSH Terms]) OR ("Bangladesh"[Text Word])) OR ("Bangladeshi"[Text Word])) OR ("Bangladesh Health System"[Text Word])) OR ("Bangladesh"[Affiliation])) AND ((((((((((("Public Health"[MeSH Terms]) OR ("Public Health"[Text Word])) OR ("Population Health"[Text Word])) OR ("Community Health"[Text Word])) OR ("Epidemiology"[Text Word])) OR	

		("Healthcare "[Text Word])) OR ("Disease Prevention"[Text Word])) OR ("Medical Care "[Text Word])) OR ("Public Health Informatics"[Text Word])) OR ("Public Health Surveillance "[MeSH Terms])) OR ("Public Health Informatics"[MeSH Terms])))	

Search Summary-2

Name of the database: Scopus

Date of search: 10th September, 2023

Timeline: 1st January, 2000 to 10th September, 2023

Search Language

1	Artificial Intelligence	TITLE-ABS-KEY ("Artificial Intelligence")	505,494
2		TITLE-ABS-KEY (ai)	168,840
3		TITLE-ABS-KEY ("machine intelligence")	3,339
4		TITLE-ABS-KEY ("cognitive computing")	1,602
5		TITLE-ABS-KEY ("intelligent system")	154,768
6		TITLE-ABS-KEY ("automated reasoning")	2,923
7		TITLE-ABS-KEY ("computational intelligence")	11,433
8		TITLE-ABS-KEY ("computer reasoning")	56
9		TITLE-ABS-KEY ("computer vision system")	4,643
10		(TITLE-ABS-KEY ("ARTIFICIAL INTELLIGENCE")) OR (TITLE-ABS-KEY (AI)) OR (TITLE-ABS-KEY ("MACHINE INTELLIGENCE")) OR (TITLE-ABS-KEY ("COGNITIVE COMPUTING")) OR (TITLE-ABS-KEY ("INTELLIGENT SYSTEM")) OR (TITLE-ABS-KEY ("AUTOMATED REASONING")) OR (TITLE-ABS-KEY ("COMPUTATIONAL INTELLIGENCE")) OR (TITLE-ABS-KEY ("COMPUTER REASONING")) OR (TITLE-ABS-KEY ("COMPUTER VISION SYSTEM"))	761,945
11	Machine Learning	TITLE-ABS-KEY ("machine learning")	519,252
12		TITLE-ABS-KEY ("transfer learning")	37,140
13		TITLE-ABS-KEY ("statistical learning")	9,803
14		TITLE-ABS-KEY ("predictive modeling")	19,671
15		TITLE-ABS-KEY ("pattern recognition")	253,034
16		TITLE-ABS-KEY ("unsupervised learning")	23,869
17		TITLE-ABS-KEY ("supervised learning")	60,427

18		TITLE-ABS-KEY ("pattern recognition system")	19,208
19		TITLE-ABS-KEY ("deep neural learning")	90
20		(TITLE-ABS-KEY ("MACHINE LEARNING")) OR (TITLE-ABS-KEY ("TRANSFER LEARNING")) OR (TITLE-ABS-KEY ("STATISTICAL LEARNING")) OR (TITLE-ABS-KEY ("PREDICTIVE MODELING")) OR (TITLE-ABS-KEY ("PATTERN RECOGNITION")) OR (TITLE-ABS-KEY ("UNSUPERVISED LEARNING")) OR (TITLE-ABS-KEY ("SUPERVISED LEARNING")) OR (TITLE-ABS-KEY ("PATTERN RECOGNITION SYSTEM")) OR (TITLE-ABS-KEY ("DEEP NEURAL LEARNING"))	855,014
21	Deep Learning	TITLE-ABS-KEY ("deep learning")	328,700
22		TITLE-ABS-KEY ("neural network")	876,082
23		TITLE-ABS-KEY ("artificial neural network")	209,716
24		TITLE-ABS-KEY ("deep neural network")	105,104
25		TITLE-ABS-KEY ("hierarchical learning")	893
26		(TITLE-ABS-KEY ("DEEP LEARNING")) OR (TITLE-ABS-KEY ("NEURAL NETWORK")) OR (TITLE-ABS-KEY ("ARTIFICIAL NEURAL NETWORK")) OR (TITLE-ABS-KEY ("DEEP NEURAL NETWORK")) OR (TITLE-ABS-KEY ("HIERARCHICAL LEARNING"))	1,040,583
27	Big Data	TITLE-ABS-KEY ("big data")	150,896
28		TITLE-ABS-KEY ("data mining")	222,214
29		TITLE-ABS-KEY ("big data analytics")	12,387
30		TITLE-ABS-KEY ("data science")	20,722
31		TITLE-ABS-KEY ("predictive analytics")	29,022
32		TITLE-ABS-KEY ("data-driven decision making")	1,909
33		TITLE-ABS-KEY ("large-scale data analysis")	713
34		(TITLE-ABS-KEY ("BIG DATA")) OR (TITLE-ABS-KEY ("DATA MINING")) OR (TITLE-ABS-KEY ("BIG DATA ANALYTICS")) OR (TITLE-ABS-KEY ("DATA SCIENCE")) OR (TITLE-ABS-KEY ("PREDICTIVE ANALYTICS")) OR (TITLE-ABS-KEY (	398,749

		"DATA-DRIVEN DECISION MAKING")) OR (TITLE-ABS-KEY ("LARGE-SCALE DATA ANALYSIS"))	
35	Healthcare System	TITLE-ABS-KEY ("public health")	710,428
36		TITLE-ABS-KEY ("population health")	25,592
37		TITLE-ABS-KEY ("community health")	100,746
38		TITLE-ABS-KEY ("epidemiology")	744,080
39		TITLE-ABS-KEY (" Healthcare ")	156,147
40		TITLE-ABS-KEY ("disease prevention")	30,346
41		TITLE-ABS-KEY (" Medical Care ")	8,756
42		TITLE-ABS-KEY ("public health informatics")	1,493
43		(TITLE-ABS-KEY ("PUBLIC HEALTH INFORMATICS")) OR (TITLE-ABS-KEY (" MEDICAL CARE ")) OR (TITLE-ABS-KEY ("DISEASE PREVENTION")) OR (TITLE-ABS-KEY ("HEALTHCARE")) OR (TITLE-ABS-KEY ("EPIDEMIOLOGY")) OR (TITLE-ABS-KEY ("COMMUNITY HEALTH")) OR (TITLE-ABS-KEY ("POPULATION HEALTH")) OR (TITLE-ABS-KEY ("PUBLIC HEALTH"))	1,609,341
44			
45	Bangladesh	TITLE-ABS-KEY (bangladesh)	59,038
46		TITLE-ABS-KEY (bangladeshi)	8,177
47		TITLE-ABS-KEY ("bangladesh health system")	15
48		AFFILCOUNTRY (BANGLADESH)	108,330
49		(TITLE-ABS-KEY (BANGLADESH)) OR (TITLE-ABS-KEY (BANGLADESHI)) OR (TITLE-ABS-KEY ("BANGLADESH HEALTH SYSTEM")) OR (AFFILCOUNTRY (BANGLADESH))	134,837
50		10 OR 20 OR 26 OR 34<br>((TITLE-ABS-KEY ("Artificial Intelligence")) OR (TITLE-ABS-KEY (ai)) OR (TITLE-ABS-KEY ("machine intelligence")) OR (TITLE-ABS-KEY ("cognitive computing")) OR (TITLE-ABS-KEY ("intelligent system")) OR (TITLE-ABS-KEY ("automated reasoning")) OR (TITLE-ABS-KEY ("computational intelligence")) OR (TITLE-ABS-KEY ("computer reasoning"))	2,485,272

		OR (TITLE-ABS-KEY ("computer vision system"))) OR ((TITLE-ABS-KEY ("machine learning")) OR (TITLE-ABS-KEY ("transfer learning")) OR (TITLE-ABS-KEY ("statistical learning")) OR (TITLE-ABS-KEY ("predictive modeling")) OR (TITLE-ABS-KEY ("pattern recognition")) OR (TITLE-ABS-KEY ("unsupervised learning")) OR (TITLE-ABS-KEY ("supervised learning")) OR (TITLE-ABS-KEY ("pattern recognition system")) OR (TITLE-ABS-KEY ("deep neural learning"))) OR ((TITLE-ABS-KEY ("deep learning")) OR (TITLE-ABS-KEY ("neural network")) OR (TITLE-ABS-KEY ("artificial neural network")) OR (TITLE-ABS-KEY ("deep neural network")) OR (TITLE-ABS-KEY ("hierarchical learning"))) OR ((TITLE-ABS-KEY ("big data")) OR (TITLE-ABS-KEY ("data mining")) OR (TITLE-ABS-KEY ("big data analytics")) OR (TITLE-ABS-KEY ("data science")) OR (TITLE-ABS-KEY ("predictive analytics")) OR (TITLE-ABS-KEY ("data-driven decision making")) OR (TITLE-ABS-KEY ("large-scale data analysis")))	
21		43 AND 49<br>((TITLE-ABS-KEY ("public health informatics")) OR (TITLE-ABS-KEY (" Medical Care ")) OR (TITLE-ABS-KEY ("disease prevention")) OR (TITLE-ABS-KEY ("healthcare")) OR (TITLE-ABS-KEY ("epidemiology")) OR (TITLE-ABS-KEY ("community health")) OR (TITLE-ABS-KEY ("population health")) OR (TITLE-ABS-KEY ("public health"))) AND ((TITLE-ABS-KEY (bangladesh)) OR (TITLE-ABS-KEY (bangladeshi)) OR (TITLE-ABS-KEY ("bangladesh health system")) OR (AFFILCOUNTRY (bangladesh)))	8,443
52		(10 OR 20 OR 26 OR 34) AND (43 AND 49)<br><br>(((TITLE-ABS-KEY ("ARTIFICIAL INTELLIGENCE")) OR (TITLE-ABS-KEY (AI	198

		)) OR (TITLE-ABS-KEY ("MACHINE INTELLIGENCE")) OR (TITLE-ABS-KEY ("COGNITIVE COMPUTING")) OR (TITLE-ABS-KEY ("INTELLIGENT SYSTEM")) OR (TITLE-ABS-KEY ("AUTOMATED REASONING")) OR (TITLE-ABS-KEY ("COMPUTATIONAL INTELLIGENCE")) OR (TITLE-ABS-KEY ("COMPUTER REASONING")) OR (TITLE-ABS-KEY ("COMPUTER VISION SYSTEM"))) OR ((TITLE-ABS-KEY ("MACHINE LEARNING")) OR (TITLE-ABS-KEY ("TRANSFER LEARNING")) OR (TITLE-ABS-KEY ("STATISTICAL LEARNING")) OR (TITLE-ABS-KEY ("PREDICTIVE MODELING")) OR (TITLE-ABS-KEY ("PATTERN RECOGNITION")) OR (TITLE-ABS-KEY ("UNSUPERVISED LEARNING")) OR (TITLE-ABS-KEY ("SUPERVISED LEARNING")) OR (TITLE-ABS-KEY ("PATTERN RECOGNITION SYSTEM")) OR (TITLE-ABS-KEY ("DEEP NEURAL LEARNING"))) OR ((TITLE-ABS-KEY ("DEEP LEARNING")) OR (TITLE-ABS-KEY ("NEURAL NETWORK")) OR (TITLE-ABS-KEY ("ARTIFICIAL NEURAL NETWORK")) OR (TITLE-ABS-KEY ("DEEP NEURAL NETWORK")) OR (TITLE-ABS-KEY ("HIERARCHICAL LEARNING"))) OR ((TITLE-ABS-KEY ("BIG DATA")) OR (TITLE-ABS-KEY ("DATA MINING")) OR (TITLE-ABS-KEY ("BIG DATA ANALYTICS")) OR (TITLE-ABS-KEY ("DATA SCIENCE")) OR (TITLE-ABS-KEY ("PREDICTIVE ANALYTICS")) OR (TITLE-ABS-KEY ("DATA-DRIVEN DECISION MAKING")) OR (TITLE-ABS-KEY ("LARGE-SCALE DATA ANALYSIS"))))) AND (((TITLE-ABS-KEY ("PUBLIC HEALTH INFORMATICS")) OR (TITLE-ABS-KEY ("MEDICAL CARE")) OR (TITLE-ABS-KEY ("DISEASE PREVENTION")) OR (TITLE-ABS-KEY ("HEALTHCARE")) OR (TITLE-ABS-KEY ("EPIDEMIOLOGY")) OR (TITLE-ABS-KEY ("COMMUNITY	

		HEALTH")) OR (TITLE-ABS-KEY ("POPULATION HEALTH")) OR (TITLE-ABS-KEY ("PUBLIC HEALTH"))) AND ((TITLE-ABS-KEY (BANGLADESH)) OR (TITLE-ABS-KEY (BANGLADESHI)) OR (TITLE-ABS-KEY ("BANGLADESH HEALTH SYSTEM")) OR (AFFILCOUNTRY (BANGLADESH))))	

Final search output: 198

Search Summary- 3

Name of the database: Embase

Date of search: 10th September, 2023

Timeline: 1st January, 2000 to 10th September, 2023

Search Language

1	Artificial Intelligence	'artificial intelligence'/exp OR 'artificial intelligence'	112,115
2		ai	147,319
3		'machine intelligence'/exp OR 'machine intelligence'	89,516
4		'intelligent system'	1,105
5		'automated reasoning'/exp OR 'automated reasoning'	151
6		'computational intelligence'/exp OR 'computational intelligence'	7,103
7		'computer reasoning'/exp OR 'computer reasoning'	55
8		'computer vision system'	230
9		#1 OR #2 OR #3 OR #4 OR #5 OR #6 OR #7 OR #8<br>'artificial intelligence'/exp OR 'artificial intelligence' OR ai OR 'machine intelligence'/exp OR 'machine intelligence' OR 'intelligent system' OR 'automated reasoning'/exp OR 'automated reasoning' OR 'computational intelligence'/exp OR 'computational intelligence' OR 'computer reasoning'/exp OR 'computer reasoning' OR 'computer vision system'	250,524
10	Machine Learning	'machine learning'/exp OR 'machine learning'	430,577

11		'transfer learning'/exp OR 'transfer learning'	6,975
12		'statistical learning'/exp OR 'statistical learning'	2,507
13		'predictive modeling'/exp OR 'predictive modeling'	14,161
14		'pattern recognition'/exp OR 'pattern recognition'	110,830
15		'unsupervised learning'/exp OR 'unsupervised learning'	3,044
16		'supervised learning'/exp OR 'supervised learning'	6,475
17		'pattern recognition system'	262
18		'deep neural learning'	18
19		#10 OR #11 OR #12 OR #13 OR #14 OR #15 OR #16 OR #17 OR #18<br>'machine learning'/exp OR 'machine learning' OR 'transfer learning'/exp OR 'transfer learning' OR 'statistical learning'/exp OR 'statistical learning' OR 'predictive modeling'/exp OR 'predictive modeling' OR 'pattern recognition'/exp OR 'pattern recognition' OR 'unsupervised learning'/exp OR 'unsupervised learning' OR 'supervised learning'/exp OR 'supervised learning' OR 'pattern recognition system' OR 'deep neural learning'	509,537
20	Deep Learning	'deep learning'/exp OR 'deep learning'	64,418
21		'neural network'/exp OR 'neural network'	120,586
22		'artificial neural network'/exp OR 'artificial neural network'	96,230

23		'deep neural network'/exp OR 'deep neural network'	34,186
24		'hierarchical learning'/exp OR 'hierarchical learning'	43,973
25		#20 OR #21 OR #22 OR #23 OR #24<br><br>'deep learning'/exp OR 'deep learning' OR 'neural network'/exp OR 'neural network' OR 'artificial neural network'/exp OR 'artificial neural network' OR 'deep neural network'/exp OR 'deep neural network' OR 'hierarchical learning'/exp OR 'hierarchical learning'	160,270
26	Big Data	'big data'/exp OR 'big data'	30,074
27		'data mining'/exp OR 'data mining'	28,026
28		'big data analytics'	1,454
29		'data science'/exp OR 'data science'	32,084
30		'predictive analytics'/exp OR 'predictive analytics'	1,476
31		'data-driven decision making'	237
32		'large-scale data analysis'	203
33		#26 OR #27 OR #28 OR #29 OR #30 OR #31 OR #32<br>'big data'/exp OR 'big data' OR 'data mining'/exp OR 'data mining' OR 'big data analytics' OR 'data science'/exp OR 'data science' OR 'predictive analytics'/exp OR 'predictive analytics' OR 'data-driven decision making' OR 'large-scale data analysis'	88,445
34	Healthcare System	'public health'/exp OR 'public health'	1,817,919

35		'population health'/exp OR 'population health'	140,612
36		'community health'/exp OR 'community health'	415,809
37		'epidemiology'/exp OR 'epidemiology'	5,633,268
38		'healthcare'/exp OR 'health promotion'	184,694
39		'disease prevention'/exp OR 'disease prevention'	1,279,331
40		' medical care /exp OR medical care '	5,024
41		'public health informatics'/de OR 'public health informatics'	23,825
42		#34 OR #35 OR #36 OR #37 OR #38 OR #39 OR #40 OR #41 'public health'/exp OR 'public health' OR 'population health'/exp OR 'population health' OR ' healthcare' /exp OR 'community health' OR 'epidemiology'/exp OR 'epidemiology' OR 'health promotion'/exp OR 'health promotion' OR 'disease prevention'/exp OR 'disease prevention' OR medical care '/exp OR medical care ' OR 'public health informatics'/de OR 'public health informatics'	7,667,853
43	Bangladesh	'bangladesh'/exp OR 'bangladesh'	51,785
44		'bangladeshi'/exp OR 'bangladeshi'	5,352
45		'bangladesh':ff	12,164
46		'bangladesh health system'	9
47		#43 OR #44 OR #45 OR #46	52,954

		'bangladesh'/exp OR 'bangladesh' OR 'bangladeshi'/exp OR 'bangladeshi' OR 'bangladesh':ff OR 'bangladesh health system'	
48		#9 OR #19 OR #25 OR #33<br>'artificial intelligence'/exp OR 'artificial intelligence' OR ai OR 'machine intelligence'/exp OR 'machine intelligence' OR 'intelligent system' OR 'automated reasoning'/exp OR 'automated reasoning' OR 'computational intelligence'/exp OR 'computational intelligence' OR 'computer reasoning'/exp OR 'computer reasoning' OR 'computer vision system' OR 'machine learning'/exp OR 'machine learning' OR 'transfer learning'/exp OR 'transfer learning' OR 'statistical learning'/exp OR 'statistical learning' OR 'predictive modeling'/exp OR 'predictive modeling' OR 'pattern recognition'/exp OR 'pattern recognition' OR 'unsupervised learning'/exp OR 'unsupervised learning' OR 'supervised learning'/exp OR 'supervised learning' OR 'pattern recognition system' OR 'deep neural learning' OR 'deep learning'/exp OR 'deep learning' OR 'neural network'/exp OR 'neural network' OR 'artificial neural network'/exp OR 'artificial neural network' OR 'deep neural network'/exp OR 'deep neural network' OR 'hierarchical learning'/exp OR 'hierarchical learning' OR 'big data'/exp OR 'big data' OR 'data mining'/exp OR 'data mining' OR 'big data analytics' OR 'data science'/exp OR 'data science' OR 'predictive analytics'/exp OR 'predictive analytics' OR 'data-driven decision making' OR 'large-scale data analysis'	755,772
49		#42 AND #47	25,716

		('public health'/exp OR 'public health' OR 'population health'/exp OR 'healthcare' OR 'community health'/exp OR 'community health' OR 'epidemiology'/exp OR 'epidemiology' OR 'health promotion'/exp OR 'health promotion' OR 'disease prevention'/exp OR 'disease prevention' OR ‘medical care '/exp OR ‘medical care’ OR 'public health informatics'/de OR 'public health informatics') AND ('bangladesh'/exp OR 'bangladesh' OR 'bangladeshi'/exp OR 'bangladeshi' OR 'bangladesh':ff OR 'bangladesh health system')	
50		#48 AND #49<br>('artificial intelligence'/exp OR 'artificial intelligence' OR ai OR 'machine intelligence'/exp OR 'machine intelligence' OR 'intelligent system' OR 'automated reasoning'/exp OR 'automated reasoning' OR 'computational intelligence'/exp OR 'computational intelligence' OR 'computer reasoning'/exp OR 'computer reasoning' OR 'computer vision system' OR 'machine learning'/exp OR 'machine learning' OR 'transfer learning'/exp OR 'transfer learning' OR 'statistical learning'/exp OR 'statistical learning' OR 'predictive modeling'/exp OR 'predictive modeling' OR 'pattern recognition'/exp OR 'pattern recognition' OR 'unsupervised learning'/exp OR 'unsupervised learning' OR 'supervised learning'/exp OR 'supervised learning' OR 'pattern recognition system' OR 'deep neural learning' OR 'deep learning'/exp OR 'deep learning' OR 'neural network'/exp OR 'neural network' OR 'artificial neural network'/exp OR 'artificial neural network' OR 'deep neural network'/exp OR 'deep neural network' OR 'hierarchical learning'/exp OR	725

		'hierarchical learning' OR 'big data'/exp OR 'big data' OR 'data mining'/exp OR 'data mining' OR 'big data analytics' OR 'data science'/exp OR 'data science' OR 'predictive analytics'/exp OR 'predictive analytics' OR 'data-driven decision making' OR 'large-scale data analysis') AND ('public health'/exp OR 'public health' OR 'population health'/exp OR 'population health' OR 'community health'/exp OR 'community health' OR 'epidemiology'/exp OR 'epidemiology' OR 'healthcare/exp OR 'healthcare' OR 'disease prevention'/exp OR 'disease prevention' OR 'medical care' /exp OR 'medical care' OR 'public health informatics'/de OR 'public health informatics') AND ('bangladesh'/exp OR 'bangladesh' OR 'bangladeshi'/exp OR 'bangladeshi' OR 'bangladesh':ff OR 'bangladesh health system')	

Final search output: 725

Search Summary-4

Name of the database: IEEE Explore (Advanced)

Date of search: 10th September, 2023

Timeline: 1st January, 2000 to 10th September, 2023

Search Language

1	Artificial Intelligence	("Full Text & Metadata":"Artificial Intelligence")	543692
2		("Full Text & Metadata":AI)	363205
3		("Full Text & Metadata":"Machine Intelligence")	41039
4		("Full Text & Metadata":"Computational Intelligence")	79913
5		(((((Full Text & Metadata:"Artificial Intelligence"))) OR ((Full Text & Metadata:"AI"))) OR ((Full Text & Metadata:"Machine Intelligence"))) OR ((Full Text & Metadata:"Computational Intelligence"))	867135
6	Machine Learning	("Full Text & Metadata":"Machine Learning")	442139
7		("Full Text & Metadata":"Pattern Recognition")	207620
8		("Full Text & Metadata":"Unsupervised Learning")	62030
9		("Full Text & Metadata":"Supervised Learning")	111227
10		(((((Full Text & Metadata:"Machine Learning"))) OR ((Full Text & Metadata:"Pattern Recognition"))) OR ((Full Text & Metadata:"Unsupervised Learning"))) OR ((Full Text & Metadata:"Supervised Learning"))	644041
11	Deep Learning	("Full Text & Metadata":"Deep Learning")	268818
12		("Full Text & Metadata":"Neural Network*")	654138
13		("Full Text & Metadata":"Artificial Neural Network*")	159297
14		("Full Text & Metadata":"Deep Neural Network*")	121075
15		(((((Full Text & Metadata:"Deep Learning"))) OR ((Full Text & Metadata:"Neural Network*"))) OR	702352

		((Full Text & Metadata:"Artificial Neural Network*"))) OR ((Full Text & Metadata:"Deep Neural Network*"))	
16	Big Data	("Full Text & Metadata":"Big Data")	147455
17		("Full Text & Metadata":"Data Mining")	210193
18		(((Full Text & Metadata:"Data Mining"))) OR ((Full Text & Metadata:"Big Data"))	323661
19	Healthcare System	("Full Text & Metadata":"Public Health*")	28104
20		("Full Text & Metadata":"Healthcare ")	7324
21		(((Full Text & Metadata:"Public Health"))) OR ((Full Text & Metadata:" Healthcare "))	33584
22	Bangladesh	("Full Text & Metadata":"Bangladesh")	26133
23		(("Full Text & Metadata":"Public Health*" OR "Full Text & Metadata":"Epidemiology") AND "Full Text & Metadata":"Bangladesh")	803
24		(("Full Text & Metadata":"Deep Learning" OR "Full Text & Metadata":"Neural Network*" OR "Full Text & Metadata":"Artificial Neural Network*" OR "Deep Neural Network*") OR (Full Text & Metadata:"Artificial Intelligence" OR Full Text & Metadata:"AI" OR Full Text & Metadata:"Machine Intelligence" OR Full Text & Metadata:"Computational Intelligence") OR ("Full Text & Metadata":"Machine Learning" OR "Full Text & Metadata":"Pattern Recognition" OR "Full Text & Metadata":"Unsupervised Learning" OR "Full Text & Metadata":"Supervised Learning") OR (Full Text & Metadata:"Data Mining" OR Full Text & Metadata:"Big Data"))	993503
25		((("Full Text & Metadata":"Deep Learning" OR "Full Text & Metadata":"Neural Network*" OR "Full Text & Metadata":"Artificial Neural Network*" OR "Deep Neural Network*") OR (Full Text & Metadata:"Artificial Intelligence" OR Full Text & Metadata:"AI" OR Full Text & Metadata:"Machine Intelligence" OR Full Text &	410

		Metadata:"Computational Intelligence") OR ("Full Text & Metadata":"Machine Learning" OR "Full Text & Metadata":"Pattern Recognition" OR "Full Text & Metadata":"Unsupervised Learning" OR "Full Text & Metadata":"Supervised Learning") OR (Full Text & Metadata:"Data Mining" OR Full Text & Metadata:"Big Data")) AND (("Full Text & Metadata":"Public Health*" OR "Full Text & Metadata":"Healthcare ") AND "Full Text & Metadata":"Bangladesh"))	

Final search output: 410
