# Supplementary material for: Implications of Big Data Analytics, AI, Machine Learning, and Deep Learning in the Health Care System of Bangladesh: Scoping Review
Source: J Med Internet Res. 2024 Oct 28;26:e54710. doi: 10.2196/54710 (PMC11555453; doi:10.2196/54710)
Supplement: Multimedia Appendix 3 [file jmir_v26i1e54710_app3.docx]

**Appendix C. Quality assessment**

**Quality assessment of Case-control Study**

**Quality assessment of Cohort Study**

**Quality assessment of Retrospective Study**

**Quality assessment of Cross-sectional Study**
